# Nucleocytoplasmic Shuttling of Viral Proteins in Borna Disease Virus Infection

**DOI:** 10.3390/v5081978

**Published:** 2013-08-08

**Authors:** Tomoyuki Honda, Keizo Tomonaga

**Affiliations:** Department of Viral Oncology, Institute for Virus Research, Kyoto University, Sakyo-ku, Kyoto 606-8507, Japan; E-Mail: tomonaga@virus.kyoto-u.ac.jp

**Keywords:** Borna disease virus, RNP, NLS, NES, replication

## Abstract

Nuclear import and export of viral RNA and proteins are critical to the replication cycle of viruses that replicate in the nucleus. Borna disease virus (BDV) is a nonsegmented, negative-strand RNA virus that belongs to the order *Mononegavirales*. BDV has several distinguishing features, one of the most striking being the site of its replication. BDV RNA is transcribed and replicated in the nucleus, while most other negative-strand RNA viruses replicate in the cytoplasm. Therefore, the nucleocytoplasmic trafficking of BDV macromolecules plays a key role in virus replication. Growing evidence indicates that several BDV proteins, including the nucleoprotein, phosphoprotein, protein X and large protein, contribute to the nucleocytoplasmic trafficking of BDV ribonucleoprotein (RNP). The directional control of BDV RNP trafficking is likely determined by the ratios of and interactions between the nuclear localization signals and nuclear export signals in the RNP. In this review, we present a comprehensive view of several unique mechanisms that BDV has developed to control its RNP trafficking and discuss the significance of BDV RNP trafficking in the replication cycle of BDV.

## 1. Introduction

Borna disease virus (BDV) is the member of the *Bornaviridae* family within the non-segmented negative-strand RNA viruses, *Mononegavirales*. BDV is highly neurotropic and establishes noncytopathic persistent infections in a wide variety of host species. BDV persistent infection causes autism-like neurobehavioral disorders, such as anxiety, aggression, hyperactivity, abnormal play behavior and cognitive deficits in various vertebrate species [1,2]. BDV, unlike most other RNA viruses, replicates in the nuclei of infected cells. Therefore, the nucleocytoplasmic trafficking of BDV proteins must play critical roles in the BDV replication cycle.

BDV is composed of five structural proteins, nucleoprotein (N), phosphoprotein (P), matrix protein (M), glycoprotein (G), and large protein (L), and one non-structural protein, X. Among these structural proteins, N, P, and L are essential for viral replication and transcription. BDV genomic RNA (gRNA) is packaged into ribonucleoprotein (RNP) complexes [3,4], which also contain N and viral RNA-dependent RNA polymerase (RdRp) complex. The RdRp complex consists of P and L and is responsible for replication and transcription of the viral genome. X is a non-structural protein with strong inhibitory activity against BDV RdRp [5,6]. M plays a critical role in virus particle assembly and budding [7]. BDV enters its target cells using the surface G protein and the expression and correct processing of G are required for efficient viral dissemination in neurons [8]. BDV neurotropism is determined by the interaction of G with its receptor [8,9] and the interaction of RNP components with host factors [10]. Transcription of BDV gRNA is believed to occur sequentially, resulting in a transcriptional gradient that is less pronounced than in most other *Mononegavirales*.

Many excellent studies have been published on the nucleocytoplasmic shuttling of BDV proteins but the implications of this process for the viral replication cycle have not been reviewed in a comprehensive manner. Therefore, in this review, we will first focus our attention on the nucleocytoplasmic shuttling of each viral protein. Then, we will discuss the implication of nucleocytoplasmic shuttling for three different stages of BDV infection: nuclear import of viral RNPs, replication of RNPs in the nucleus, and egress of nascent RNPs from the nucleus. Because BDV has evolved several unique strategies to exploit the host nuclear transport system, reviewing these strategies may shed light on the BDV replication cycle and point the way to novel strategies to control virus replication in other viral infections.

## 2. Nucleocytoplasmic Shuttling of BDV Proteins

To enable molecules to cross the nuclear membrane, cells have developed an elegant transport system using the importin-β superfamily [11]. Most members of this superfamily bind cargo molecules containing a nuclear localization signal (NLS) and/or nuclear export signal (NES), which function independently of the surrounding sequences [12,13]. Directional transport is maintained through association with the small GTPase protein, Ran [14]. Importins bind cargo molecules with an NLS in the cytosol and release them on binding the GTP-bound form of Ran in the nucleus. Conversely, exportins (such as chromosome region maintenance protein 1, CRM1), which also belong to the importin-β superfamily, bind cargo molecules with an NES in the nucleus and release them when Ran hydrolyzes GTP to GDP in the cytosol [14].

Similar to other viruses, BDV encodes proteins with critical functions in the nucleocytoplasmic transport of BDV RNP [15,16,17]. In this section, we provide an overview of the nucleocytoplasmic shuttling of each BDV protein, focusing on its NLS or NES and protein-protein interactions.

### 2.1. N Protein

**Figure 1:**
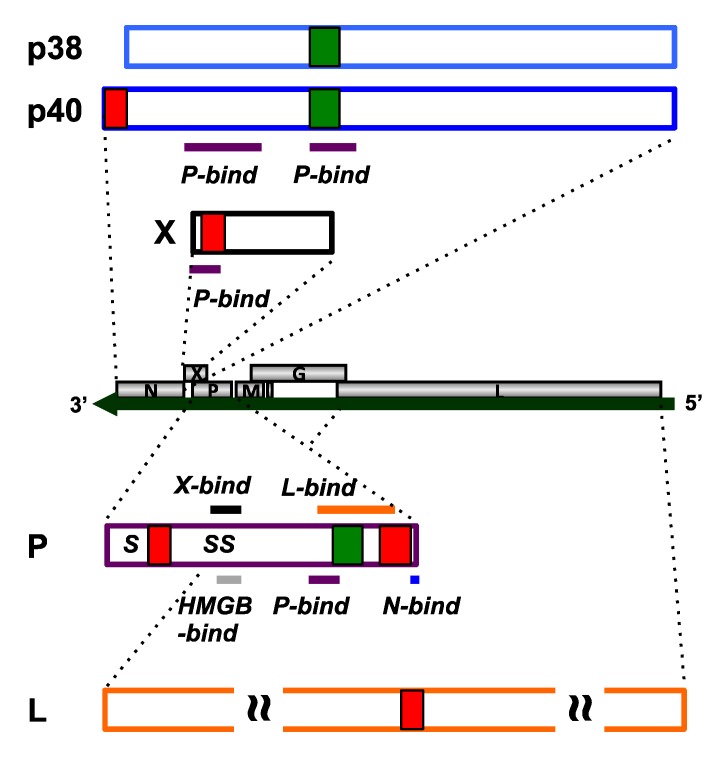
Map of nuclear transport signals in the BDV proteins and sites of interaction with other proteins. The red boxes indicate NLSs and the green boxes indicate NESs. “S” in P indicates the site of phosphorylation.

N is the major essential component of BDV RNP and encapsidates BDV gRNA. There are two isoforms of N, p40 and p38, translated from the first AUG and second AUG of N mRNA, respectively [18]. Both N proteins are found in the nucleus and cytoplasm of BDV-infected cells [18]. When p40 is expressed alone, it is localized in the nucleus [19]. On the other hand, when p38 is expressed alone, it is localized in the cytosol [20]. The difference in the subcellular localization of p40 and p38 can be explained by their NLS and NES (Figure 1). The NLS of p40 is located in the N‑terminus (P_3_KRRLVDDA_11_), which is not present in p38 [19]. p40 also has a canonical, leucine-based NES (L_128_TELEISSIFSHCC_141_), which is also present in p38 [20]. The nuclear export of N is via a CRM1-dependent pathway, consistent with the presence of this NES [20]. Using the NLS and NES, p40 shuttles between the nucleus and the cytosol. On the other hand, p38 is localized in the cytosol because it lacks an NLS. When p40 and p38 are expressed simultaneously, both proteins are found in both the nucleus and the cytosol [20]. Because N forms a planar homo-tetramer and p40 and p38 interact with each other, p40 efficiently promotes the nuclear import of p38 via interaction with these two N proteins [4,20].

N interacts with another BDV protein, P. Interaction with P is facilitated by two sites located in the N-terminal half of N (aa 51–100 and 131–158 in p40) [21]. The latter P-binding site overlaps with the NES of N. Because of this overlap, the interaction of N with P can prevent the nuclear export of N by masking of its NES [20]. P also contains NLSs (see below) and these also can affect the subcellular localization of N.

### 2.2. P Protein

P is a multifunctional BDV protein and an essential component of BDV RNP. It plays critical roles as a co-transcriptional factor of L and in encapsidation of the gRNA [3]. Similar to other viral phosphoproteins, P is phosphorylated at serine residues *in vivo* [22]. The major phosphorylation site is located at S_28_ (and S_26_ in some strains) for protein kinase Cε and there are minor sites at S_70_ and S_86_ for casein kinase II (CKII). However, in contrast to other viral phosphoproteins, the co-factor activity of P for BDV RdRp is negatively regulated by phosphorylation [23]. P is efficiently transported to the nucleus and is localized in the nuclei of transgenic mice expressing P selectively in astrocytes [24,25]. P contains two NLSs at the N- and C-termini (P_29_RPRKIPR_36_ and P_181_PRIYPQLPSAPT_193_, respectively) [26,27]. P has a unique CRM1-dependent NES within its methionine-rich domain (M_145_KTMMETMKLMMEKVDLLYAS_165_), which contains six methionine and three leucine residues [16].

P serves as a molecular hub for the BDV proteins (Figure 1). It interacts with N through its C‑terminal part (aa 197–201) [3]. It also interacts with X through the region (aa 72–87), which overlaps with the binding region (aa 77–86) of a host protein, high mobility group box 1 (HMGB1), and the phosphorylation sites for CKII [3]. When X and P are expressed simultaneously, P is efficiently localized in the cytosol [17]. Phosphorylation of P does not affect its subcellular localization. However, substitutions of alanine for serine at the CKII phosphorylation sites weaken the interaction of P with X, resulting in nuclear retention of P in the presence of X [17]. P forms homo‑oligomers through the region (aa 135–172), which overlaps with the region that binds L (aa 135–183) and the methionine-rich NES [3,16,28]. Oligomerization of P is essential for BDV polymerase activity [28]. Substitutions of alanine for methionine dramatically impairs the methionine-rich NES-mediated nuclear export of P in the presence of X [16].

### 2.3. X Protein

X is a negative regulator of BDV polymerase but is essential for virus propagation [5,29,30]. It is localized in both the nucleus and the cytosol [31] and also is localized in viral nuclear speckles in BDV-infected cells, similar to other BDV RNP components. X has an unusual NLS, with a similar sequence to a leucine-rich NES, in the N-terminus (R_6_LTLLELVRRNGN_19_) (Figure 1) [32]. The nuclear import of X is mediated through the binding of its NLS with importin-α [32]. Although this sequence is a putative NES, it does not have nuclear export activity.

X is associated with P as described above. The site of interaction with P maps to the N-terminal region (aa 3–16) of X [33,34]. The P-binding site overlaps with the NLS of X. Interaction between X and P facilitates nuclear export of P [16,17]. Indeed, P is efficiently retained in the cytosol of BDV‑infected cells only when the expression of X is detectable [17]. X interacts with a host chaperone protein, the constitutive heat shock cognate 70 (Hsc70) [31]. The site of interaction with Hsc70 is located in the N-terminal region of X (aa 1–16) and overlaps with the P-binding site and the NLS. P interferes competitively with the binding of Hsc70 to X, which is required for the nuclear import of X [31]. Because the translation of X is suppressed in the absence of P, the expression of P precedes that of X [35]. In the early stage of BDV infection, P translocates to the nucleus via its bona fide NLS and in the absence of X. Then, X is translated and translocates to the nucleus using Hsc70. The interaction of X with P in the nucleus displaces Hsc70 from X, resulting in the nuclear export of X and P.

### 2.4. L Protein

L is a 190 kDa BDV protein containing the characteristic motifs of a viral RdRp [36]. Similar to other viral RdRp, L is phosphorylated by host cell kinases and interacts with P [37]. L is localized in the nucleus of BDV-infected cells and when it is expressed alone [37]. The NLS of L is located in the middle part (R_844_VVKLRIAP_852_) (Figure 1) [38].

## 3. Implication of Nucleocytoplasmic Shuttling for the BDV Replication Cycle

BDV enters the cells via the host endocytotic pathway [39]. When the pH in the BDV-containing endosome decreases and the endosomal and viral membranes fuse, released BDV RNPs are imported into the nucleus, where replication and transcription of the RNP occur. Upon entering the nucleus, RNPs are transcribed to produce BDV mRNA. Because replication of negative-stranded RNA viruses requires encapsidation of nascent viral gRNA by N [40], newly synthesized subunits of BDV RNP must be imported from the cytosol into the nucleus for BDV replication. After assembly, newly synthesized RNPs egress from the nucleus to propagate BDV infection to adjacent uninfected cells. In this section, we discuss the implication of nucleocytoplasmic shuttling for BDV at three different stages of the BDV replication cycle.

### 3.1. Nuclear Import of Incoming Viral RNPs

**Figure 2:**
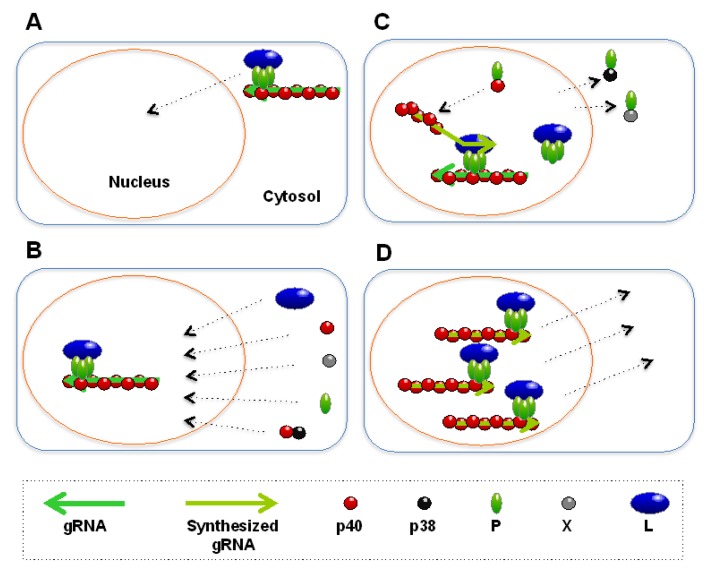
Nuclear import and export events in the BDV replication cycle. (**A**) Nuclear import of the incoming BDV RNP. BDV RNPs translocate into the nucleus via NLSs at their surface. (**B**) Nuclear import of viral RNP components after translation of BDV mRNA transcribed from BDV RNP. Most of the RNP components, except for p38, translocate into the nucleus mediated by their bona fide NLSs; p38, which lacks an NLS, translocates into the nucleus with the help of p40. (**C**) Replication of BDV RNP in the nucleus. In the nucleus, P forms two complexes: the N^0^-P and P-L complexes. Upon replication, a nascent gRNA binds to N^0^, displacing P. The stoichiometry of N-to-P is tightly regulated for efficient replication through nuclear export mediated by the binding of P with p38 and X (see Section 3.2). (**D**) Nuclear export of newly synthesized BDV RNP. The nascent RNPs are exported by unknown mechanisms, perhaps mediated by p38, P or M (see Section 3.3).

Incoming BDV RNPs contain N, P, and L. N has one NLS and one NES [19,20] but the crystal structure of N indicates its NES is usually inaccessible to solvent and buried deeply within the hydrophobic core of its N-terminal region [4]. Even in the event that the NES on N is exposed on the surface of BDV RNP following conformational changes, the NES overlaps with the P-binding site on N so that P can block its nuclear export activity [20]. Although total N is more abundant than P, N exposing its NES to the surface might be rare so that P can outcompete with this NES. P has two NLSs and one NES. The NES of P functions only in the presence of X [16]. This may be due to an X‑induced conformational change of P, which impairs the function of the P NLSs and makes the P NES dominant in the X-P complex. However, X is not incorporated into incoming RNPs and the NES of P will not function in this situation. L has only one NLS. All these factors indicate that the only accessible nuclear transport signals in incoming replication-competent RNPs are NLSs. This availability of NLSs results in efficient nuclear import of incoming BDV RNPs after their delivery to the cytosol (Figure 2A). Once in the nucleus, BDV RNPs anchor to the host chromatin through HMGB1 [10]. This, in turn, keeps BDV RNPs in the nucleus.

### 3.2. Replication and Transcription of RNPs in the Nucleus

After entering the nucleus, BDV RNPs are transcribed by its RdRp complex, and BDV mRNAs are exported to the cytosol. Then, BDV mRNAs are translated in the cytosol and the translated nascent RNP components are imported into the nucleus. The nuclear import of the RNP components may occur independently through their bona fide NLSs because all of the components are targeted efficiently to the nucleus when they are expressed alone (Figure 2B). Only p38, which lacks an NLS, is imported into the nucleus with the help of p40 (Figure 2B).

By entering the nucleus, BDV is able to benefit from its nuclear localization. One possible benefit is chaperoning BDV proteins. Molecular chaperones play important roles in virus replication, including BDV. The import machinery itself appears to play roles in chaperoning RNP subunits of influenza virus [41,42]. Thus, influenza virus, which replicates in the nucleus, appears to link chaperone functions to nuclear import pathways, making the nuclear compartment the site of assembly of correctly folded RNPs [14]. Similar to influenza virus, BDV might use chaperone activity of the import machinery. Consistent with this idea, one molecular chaperone, HSC70, is involved in both nucleocytoplasmic shuttling of X and BDV replication [31]. Further investigation is still required for confirmation of this idea.

During the assembly of new viral RNPs, two fundamentally different interactions of P with the RNP components occur in the nucleus (Figure 2C) [43]. One is the interaction between P and free N (N^0^) that is believed to prevent the aggregation of N and help to form the encapsidation complex (N^0^-P), which is essential for specific and efficient packaging of viral gRNA. The N^0^-P interaction may mask the NES in the N^0^-P complex, supporting nuclear retention of the complex until the completion of BDV gRNA encapsidation by N^0^. Although structural analysis of N tetramer reveals two possibilities for RNA binding to N, the central channel and a groove running diagonally across the tetramer surface, a major drawback of the former possibility is the need for disassembly of N to access gRNA [4]. For this reason, BDV gRNA is believed to wind around individual tetramers of N [4]. The P-binding site exposed to the solvent at the side of the N tetramer is a part of this putative RNA binding site [4]. This overlap of binding sites suggests that P and gRNA bind to N competitively, so that the interaction between N^0^ and P may be disrupted by the encapsidation of nascent gRNA. The other interaction is that between P and L, which is essential for the assembly and activity of the RdRp complex. Because N is the most abundant viral protein in infected cells, N^0^ outnumbers L. The interaction between the functional P-L complex and abundant N^0^ would sequester BDV RdRp activity. Therefore P cannot interact simultaneously with L and N^0^ for efficient replication. Indeed, the interaction between L and P is efficiently blocked in the presence of free N [43].

Because P can interact with all the components of BDV RNP as described in Section 2.2, it has a central regulatory role in BDV replication. The oligomerization activity of P is also required for efficient replication [28]. Taken together, the mode of BDV replication would fit the cartwheeling model as described previously [43,44]. During BDV transcription, the P-L complex interacts with the N-gRNA complex. The P oligomer serves as legs on which L walks along the N-gRNA complex, simultaneously making and breaking P-N contacts. Thus, the interaction of L with P may change the conformation of P, which might enhance the affinity of P with N, resulting in binding of P to the P‑binding site of N occupied by gRNA. This interaction may open the structure of the N-gRNA complex so that the RdRp can access the bases. After the transcription of the bases, L dissociates from P and then P oligomerization activity recruits new P molecule to the adjacent bases. This new P interacts with L, opening the bases and then elongating the transcription product.

Differences in the N-to-P stoichiometry are found in persistently and acutely BDV-infected cells [45]. Using a BDV minigenome assay, it has been shown that the N-to-P stoichiometry, but not the L-to-P, affects BDV RdRp activity [5]. Overexpression of p38 affects BDV RdRp activity [46]. Because p38 itself does not support BDV transcriptional activity and retains P-binding activity, it is thought to affect BDV RdRp activity by changing the relative amount of P available for BDV replication. X, a negative regulator of BDV replication, interacts preferentially with a P monomer, suggesting that X may interfere with the precise P oligomerization required for an efficient BDV replication [28]. Furthermore, X sequesters P in the cytosol, which also contributes to the negative effect of X on BDV replication [17]. Because p38 and X are nucleocytoplasmic shuttling proteins as described in section 2, the nucleocytoplasmic shuttling of these proteins is one of the strategies that BDV has evolved to control the N-to-P stoichiometry (Figure 2C). This stringent control of the N-to-P stoichiometry required for efficient replication may provide additional support for the cartwheeling model of BDV replication as described above.

In addition to the viral proteins, host proteins also regulate BDV replication in the nucleus. HMGB1, which binds to BDV RNP, is required for efficient BDV replication [10]. The interaction between HMGB1 and BDV RNP is not stable and a brief interaction is sufficient to regulate BDV replication. Because the HMGB1-binding site on P overlaps with that of X, X can affect the interaction between HMGB1 and BDV RNP, interfering with BDV replication. Hsc70, which binds to X, is also required for efficient BDV replication [31]. The enhanced interaction of X with Hsc70 results in a reduction of the binding between X and P, because P and Hsc70 share the binding site on X. This leads to an increase in the level of P in the nucleus and could affect BDV replication.

### 3.3. Egress of Nascent RNPs from the Nucleus

Newly synthesized RNPs must egress from the nucleus to produce infectious particles that can propagate BDV infection to uninfected neighboring cells (Figure 2D). The directional control of BDV RNP trafficking is determined by the ratios of and interactions between functional NLSs and NESs. As described above, RNPs are believed to expose only NLSs. This raises the question how nascent BDV RNPs translocate into the cytosol from the nucleus. We will discuss three potential mechanisms by which BDV RNPs may egress from the nucleus.

#### 3.3.1. Regulation by p38

As described in Section 2.1, in the presence of p38, p40 is located in both the nucleus and the cytosol. This property of p38 may provide a unique mechanism for the nuclear export of BDV RNPs. The presence of the NLS-lacking p38 in BDV RNPs would increase the relative number of NESs to NLSs and this may enable the nuclear export of BDV RNPs [20]. However, p38 does not support the transcription or replication of BDV [6]. Because of this feature of p38, only incompetent RNPs that do not replicate are supposed to be exported to the cytosol by this mechanism. To replicate these RNPs after infection, p38 might be replaced with p40 in the cytosol by unknown mechanism.

#### 3.3.2. Regulation by P

BDV RNP contains one NES on N and one NES on P. Binding of P with N could mask the NES on N [20]. Because the molecular ratio between the N and P proteins differs significantly between persistently and acutely BDV-infected cells [45], balanced stoichiometry of N-to-P may be required for efficient nuclear export of BDV RNPs. When N outnumbers P in the nucleus, the availability of P to mask the NES on N in BDV RNPs may be reduced, resulting in efficient nuclear export of RNPs. However, the crystal structure of N shows this NES is difficult to be accessed by other molecules, making this hypothesis unlikely [4]. X is required for the efficient methionine-rich NES‑mediated nuclear export of P by binding to P and masking its NES [16]. The expression of X may be a trigger for the nuclear export of RNPs. However, X is a non-structural protein and is not incorporated into BDV RNPs. One possible explanation for this is that X might be incorporated into BDV RNPs in the nucleus and, after nuclear export, dissociate from the RNPs in the cytosol prior to virus particle assembly.

#### 3.3.3. Regulation by M

An alternative possible mechanism involves M in the nuclear export of BDV RNPs. The matrix protein of RNA viruses is critical for viral particle assembly and budding. In addition to these functions, the matrix protein binds to viral RNP and is involved in RNP transport and replication. For example, in the case of influenza virus, which also replicates in the nucleus of infected cells, matrix (M1) protein enters the nucleus, is incorporated into viral RNPs, and is involved in the cytosolic localization of viral RNPs [47]. Similar to other viruses, BDV M plays a role in virus budding and is an integral component of viral RNPs [48,49]. When M is expressed alone, it is distributed predominantly in the nucleus. Consistent with these observations, we found a putative bipartite NLS (E_9_LKDKVIVPGWPTLMLEIDFVGGTSRNQF_37_). On the other hand, when M is expressed in BDV‑infected cells, M is localized in the viral nuclear speckles, with other viral RNP components, and in the cytosol [49]. These results suggest that M is incorporated into BDV RNP in the nucleus and may export the RNP to the cytosol. To evaluate this hypothesis, we searched for an NES in M and found a traditional conserved NES-like sequence (L_83_LLTLNSLSV_92_). This putative NES in M is located at helix α3 in the M monomer and near the outside of the M tetramer [50], suggesting that this NES may be readily accessed by other molecules. M binds to P at its N-terminus (aa 1–11), which does not overlap with the known binding sites for N, P, and L [49]. This indicates that M can bind to P without inferring with the formation of BDV RNP. Binding of P with M might expose the NES of M to the nuclear export machinery, resulting in the nuclear export of BDV RNPs. Although this hypothesis is attractive, it remains to be determined whether this putative NES on M is functional.

## 4. Perspective

In this review, we summarized briefly the nucleocytoplasmic shuttling of BDV proteins and its implication for the BDV replication cycle. In reviewing these studies, we have provided several important insights. First, although some NESs are present in BDV RNP components, only NLSs are accessible in replication-competent BDV RNPs. This may be one of the reasons why BDV remains in the nucleus for a long time and propagates to uninfected neighboring cells slowly. Second, from the accessibility of the nuclear transport signals by other molecules and the localization of BDV proteins, we can imagine the sequential interactions of BDV molecules during BDV replication, which may fit well with the cartwheeling model of viral transcription. Finally, P has a unique NES and X has an NES-like sequence that acts as an NLS rather than an NES. These sequences might shed light on the nucleocytoplasmic shuttling of host and other viral proteins. Although much remains to be determined for complete understanding of the BDV replication cycle, especially precise mechanisms how BDV RNP egresses from the nucleus, it is clear that the nucleocytoplasmic shuttling of BDV proteins plays important roles in that cycle. It may be interesting to analyze how this nucleocytoplasmic shuttling of BDV proteins is involved in BDV pathogenesis by using recombinant BDVs carrying NLS and/or NES mutations.
